# Mulberry Fruit Extract Affords Protection against Ethyl Carbamate-Induced Cytotoxicity and Oxidative Stress

**DOI:** 10.1155/2017/1594963

**Published:** 2017-07-27

**Authors:** Wei Chen, Yuting Li, Tao Bao, Vemana Gowd

**Affiliations:** Department of Food Science and Nutrition, National Engineering Laboratory of Intelligent Food Technology and Equipment, Key Laboratory for Agro-Products Postharvest Handling of Ministry of Agriculture, Zhejiang Key Laboratory for Agro-Food Processing, Fuli Institute of Food Science, Zhejiang University, Hangzhou 310058, China

## Abstract

Ethyl carbamate (EC) is a food and environmental toxicant and is a cause of concern for human exposure. Several studies indicated that EC-induced toxicity was associated with oxidative stress. Mulberry fruits are reported to have a wide range of bioactive compounds and pharmacological activities. The present study was therefore aimed to investigate the protective property of mulberry fruit extract (MFE) on EC-induced cytotoxicity and oxidative stress. Chemical composition analysis showed that total phenolic content and total flavonoid content in MFE were 502.43 ± 5.10 and 219.12 ± 4.45 mg QE/100 g FW. Cyanidin*-3-O-*glucoside and cyanidin*-3-O-*rutinoside were the major anthocyanins in MFE. *In vitro* antioxidant studies (DPPH, ABTS, and FRAP assays) jointly exhibited the potent antioxidant capacity of MFE. Further study indicated that MFE protected human liver HepG2 cells from EC-induced cytotoxicity by scavenging overproduced cellular ROS. EC treatment promoted intracellular glutathione (GSH) depletion and caused mitochondrial membrane potential (MMP) collapse, as well as mitochondrial membrane lipid peroxidation, whereas MFE pretreatment significantly inhibited GSH depletion and restored the mitochondrial membrane function. Overall, our study suggested that polyphenolic-rich MFE could afford a potent protection against EC-induced cytotoxicity and oxidative stress.

## 1. Introduction

Ethyl carbamate (EC) is largely found in various fermented food products, tobacco, and alcoholic beverages [1]. Various animal studies suggested that EC has a potential to induce cytotoxicity and genotoxicity which further leads to cancer development [2, 3]. Due to its carcinogenic nature, EC was classified as a group 2A carcinogen by the International Agency for Research on Cancer (IARC), referring to it as probably carcinogenic to humans [4]. Despite the fact that human exposure to ethyl carbamate via food and beverages is minimum, human exposure to ethyl carbamate for a long time could not be neglected.

Accumulating evidences suggested that EC-induced toxicity could be associated with oxidative stress followed by augmentation and accumulation of ROS [5]. Therefore, abrogation of oxidative stress followed by increasing antioxidant defence could be considered as a therapeutic tool in the treatment of EC-induced toxicity. Recently, many studies have focused on natural bioactive molecules present in fruits and vegetables because these natural bioactive compounds have a potential to attenuate cellular oxidative stress. For example, blackberry extract and digest are reported to protect Caco-2 cells from oxidative damage induced by ethyl carbamate [6] and blueberry fruit polyphenolics suppress oxidative stress-induced skeletal muscle cell damage *in vitro* [7]. These studies give us an idea whether dietary EC-induced cytotoxicity and oxidative stress could be ameliorated by consuming fruits and vegetables.

Mulberry is listed as a kind of homology of medicine and food in China. A large number of studies have reported that mulberry is rich in flavonoids and anthocyanins which give it a potent antioxidant activity [8, 9]. In addition, mulberry fruits have also been proved to possess various biological activity including antiobesity [10, 11], antihyperglycemic [12], and anti-inflammatory effects [13]. However, till date rare studies focus on the relationship between mulberry fruits and EC-induced cytotoxicity. Therefore, the present study was designed to investigate the effect of mulberry fruit extract (MFE) on EC-induced cytotoxicity and elucidate the protective mechanism of MFE against EC-induced oxidative stress.

## 2. Materials and Methods

### 2.1. Chemicals and Materials

Cyanidin*-3-O-*glucoside, cyanidin*-3-O-*rutinoside, pelargonidin*-3-O-*glucoside, quercetin*-3-O-*rutinoside (rutin), ascorbic acid, Folin and Ciocalteu's phenol reagent, 3-(4,5-dimethyl-2-thiazolyl)-2,5-diphenyl-2H-tetrazolium bromide (MTT), nonyl acridine orange (NAO), Hoechst 33258, 2′,7′-dichlorofluorescin diacetate (DCFH-DA), rhodamine 123 (Rh123), and Naphthalene-2,3-dicarboxaldehyde (NDA) were purchased from Sigma-Aldrich (St. Louis, MO, USA). All other reagents used were of analytical grade.

### 2.2. Mulberry Fruits

Fresh black mulberry fruits (*Morus alba* L.) were obtained from Zhejiang province, China. After obtaining the fruits, they were thoroughly washed with sterile distilled water and dried in a clean and dust-free environment under shade. For analysis, fruits were selected based on their uniformity in colour, shape, weight, and moisture content.

### 2.3. Sample Preparation

Black mulberry fruits (1 kg) chosen for analysis were homogenised and subjected to extraction followed by adding 3000 mL of ethanol/water (70 : 30, *v*/*v*) in the dark at room temperature for 1 h. The extraction procedure was repeated twice. The extracted mixture was then filtered and samples were collected. Collected samples were concentrated using rotary evaporation under reduced pressure at 40°C for experimental analysis.

### 2.4. Analysis of Phenolic Compounds

#### 2.4.1. Total Phenolic Compounds Analysis

Total phenolic content (TPC) of mulberry fruit extract (MFE) was determined by Folin-Ciocalteu method [14]. Briefly, 0.6 mL of diluted MFE (1 : 6, *v*/*v*, extract/distilled water) was added with 0.1 mL of Folin-Ciocalteu reagent and 0.2 mL of sodium carbonate solution (15%). The absorbance of the mixture was measured at 760 nm followed by 2 h incubation at room temperature. Results were expressed as mg gallic acid equivalents (GAE)/100 g fresh weight (FW) as gallic acid was used as a reference standard for the quantification of TPC.

#### 2.4.2. Total Flavonoid Analysis

Total flavonoid content (TFC) of MFE was determined by colorimetric method as previously described [14]. In brief, 0.04 mL of 5% NaNO_2_ and 0.45 mL of distilled water were added to 0.05 mL of MFE and incubated for 5 min. After incubation time, 0.04 mL of 10% Al(NO_3_)_3_ was added to the reaction mixture and incubated for 6 min. Further, 0.4 mL of 4% (*v*/*v*) NaOH and 0.02 mL distilled water were added to the reaction mixture and incubated for 15 min. Finally, absorbance was measured at 510 nm after incubation. The data was expressed as mg quercetin*-3-O-*rutinoside (rutin) equivalents (QE)/100 g fresh weight (FW) as quercetin*-3-O-*rutinoside was used as a reference standard for the quantification of TFC.

#### 2.4.3. HPLC Analysis of Anthocyanins

Anthocyanins of MFE were analysed by HPLC with a diode array detector (Dionex UltiMate 3000, ThermoFisher Scientific, USA) using Promosil C18 column (4.6 × 250 mm, 5 *μ*m). The mobile phase consisted of 1.5% formic acid in water (solvent A) and formic acid/acetonitrile/methanol/water (1.5 : 22.5 : 22.5 : 48.5, *v*/*v*/*v*/*v*) (solvent B). A linear gradient program was carried out as follows: 7 to 25% (solvent B) from 0 to 35 min, 25 to 65% (solvent B) from 35 to 45 min, 65 to 100% (solvent B) from 45 to 46 min, 100% of solvent B from 46 to 50 min, and 100 to 7% (solvent B) from 50 to 57 min followed by 7% of solvent B for 3 min. 10 *μ*L of the sample was injected, and the flow rate was set to 1.0 mL/min. Detection of anthocyanins was carried out at 520 nm. Qualitative and quantitative analysis of anthocyanins were performed according to the published data and commercial available standards (cyanidin*-3-O-*glucoside, cyanidin*-3-O-*rutinoside, and pelargonidin*-3-O-*glucoside).

### 2.5. Antioxidant Property of MFE

#### 2.5.1. Radical Scavenging Assay (DPPH)

Radical scavenging activity of MFE was evaluated using DPPH (2,2-diphenyl-1-picrylhydrazil) as a free radical according to the method previously described with slight modifications [8]. Briefly, 20 *μ*L of MFE (different concentrations) was added to 700 *μ*L of 0.1 mM DPPH ethanol solution and the mixture kept in the dark at room temperature (30 min). The absorbance was measured at 517 nm. DPPH radical scavenging rate (%) was calculated for each concentration of MFE using the following formula:

(1) $$\begin{array}{c} \%\;radical\;scavenging\;rate=\left(\frac{A_{control}-A_{sample}}{A_{control}}\right)\times 100. \end{array}$$

Vitamin C was used as a positive control. As a consequence, the DPPH radical scavenging activity of MFE was expressed as vitamin C equivalents (mg/g of fresh weight).

#### 2.5.2. ABTS Radical Scavenging Activity of MFE

The ABTS radical cation (ABTS^+^) assay method [8] adopted to evaluate the radical scavenging activity of MFE. A stable stock solution of ABTS radical cation was produced by adding 10 mL of 7 mM of 2,2-azobis (2-amidinopropane) dihydrochloride to 179 mL of 140 mM aqueous potassium persulfate, and the mixture was incubated at room temperature for 12 h in the dark. Then, the stock solution was diluted 20 times with distilled water to prepare the ABTS^+^·working solution. 20 *μ*L of MFE was added with 700 *μ*L ABTS^+^·working solution and incubated for 6 min at room temperature in the dark. The decrease in absorbance was measured at 734 nm. ABTS radical scavenging rate (%) was calculated for each concentration of MFE using formula (1). Vitamin C was used as a positive control. The ABTS radical scavenging activity of MFE was expressed as vitamin C equivalents (mg/g of fresh weight).

#### 2.5.3. Ferric Reducing Antioxidant Power (FRAP)

Ferric reducing activity of MFE was performed by FRAP assay according to the method described in the literature with slight modifications [15]. The FRAP reagent was prepared by mixing 10 mL of 10 mmol/L TPTZ solution, 10 mL of 20 mmol/L FeCl_3_, and 100 mL of 300 mmol/L acetate buffer (pH 3.5). 20 *μ*L of MFE was incubated at 37°C for 30 min followed by addition of 700 *μ*L FRAP solution, and the absorbance was measured at 593 nm. The results were expressed as vitamin C equivalents (mg/g of fresh weight).

### 2.6. Cell Culture and Treatment

Human liver HepG2 cells were obtained from the Cell Bank of Type Culture Collection of Chinese Academy of Sciences. Cells were cultured in DMEM medium (Gibco) containing 10% of the new calf serum, 100 units/mL penicillin, and 100 units/mL streptomycin. The cells were incubated in a humidified atmosphere of 5% CO_2_ at 37°C. According to different experiments, for control group, HepG2 cells were incubated in the absence of EC and MFE. For EC group, cells were incubated with EC for 24 h, whereas for treated group, cells were preincubated with MFE for 24 h, before exposure to EC for 24 h.

### 2.7. Cell Viability Assay

MTT assay was used to determine the effect of EC and MFE on cell viability according to the method described earlier [16]. HepG2 cells were seeded into 96-well cell culture plates, with the concentration of 3.5 × 10^3^ cells/well. The stock solution of EC was prepared in concentration of 1 M by dissolving it in deionized water. For the control group, HepG2 cells were incubated in the absence of EC and MFE. For the EC group, cells were incubated with EC for 24 h, whereas for treated group, the cells were preincubated with MFE for 24 h, before exposure to EC for 24 h. Subsequently, the cells were incubated with 0.5 mg/mL of MTT for 4 h. After incubation, the supernatant was removed and the formed formazan precipitate was dissolved with 150 *μ*L of DMSO. The optical density was measured at 490 nm using a Tecan infinite M200 microplate reader.

### 2.8. Measurement of Intracellular Reactive Oxygen Species (ROS)

Intracellular production of ROS was measured using the oxidation-sensitive fluorescent probe DCFH-DA as described earlier [17]. Briefly, HepG2 cells at a concentration of 2.5 × 10^4^ cells/well were seeded into 24-well cell culture plates and cultured for 24 h. Then, the cultured cells were washed with PBS and preincubated with MFE at different concentrations (0.5 mg/mL, 1 mg/mL, and 2 mg/mL) for 6 h. Subsequently, the cells were incubated with EC (60 mM) for 24 h. After MFE and EC treatment, the cells were harvested and washed with PBS then incubated with 10 *μ*M DCFH-DA at 37°C for 30 min. Cells were washed three times with PBS, and immediately, the induction of ROS was examined by fluorescence microscope. The images were considered from six different microscopic fields, and results were expressed as mean DCF fluorescence intensity calculated by image analysis software ImageProPlus6.0 (Media Cybernetics Inc.).

### 2.9. Hoechst 33258 Nuclear Staining Analysis

A fluorescence probe Hoechst 33528 was used for nuclear staining according to the method described earlier [18]. Following overnight culturing of HepG2 cells at a concentration of 2.5 × 10^4^ cells/well in a 24-well plate, the cells were treated with different concentrations of MFE (0.5 mg/mL, 1 mg/mL, and 2 mg/mL) for 24 h before they were incubated with EC (60 mM). After 24 h incubation, the cells were washed thrice with PBS and incubated with 10 *μ*M Hoechst 33258 at 37°C for 30 min in the dark. Then, the cells were washed with PBS and the cells were examined with fluorescence microscope.

### 2.10. Determination of Cellular Glutathione (GSH)

For cellular GSH detection, the HepG2 cells were treated with different concentrations of MFE (0.5 mg/mL, 1 mg/mL, and 2 mg/mL) for 24 h before they were incubated with EC (60 mM) for 24 h. Then, the cells were washed with PBS and incubated with 50 *μ*M NDA for 30 min at 37°C. Followed by incubation with fluorescence probe, the cells were washed with PBS to remove unbound fluorescence probe and then immediately evaluated by observing under fluorescence microscope.

### 2.11. Determination of Mitochondrial Membrane Potential

Mitochondrial membrane potential (MMP) was measured according to the method described earlier [19]. Briefly, HepG2 cells were incubated for 24 h with EC (60 mM) in the presence or absence of MFE (0.5 mg/mL, 1 mg/mL, and 2 mg/mL). Then, the cells were incubated at 37°C for 30 min with Rh123 (10 *μ*g/mL). Followed by incubation, the cells were washed thrice with PBS and immediately analysed under fluorescence microscope. The results were expressed as mean Rh123 fluorescence intensity.

### 2.12. Flow Cytometric Determination of Mitochondrial Membrane Potential (*Ψ*m)

MMP was further determined using flow cytometry according to the method described earlier [20]. Briefly, HepG2 cells were seeded in 6 cm cell culture dishes with a density of 1 × 10^6^ per dish for 24 h. Then, MFE (1 mg/mL and 2 mg/mL) was added to each dish for 24 h, followed by 60 mM EC for another 24 h. After being washed with PBS three times, HepG2 cells were collected by centrifugation and then incubated with Rh123 fluorescence probe for 30 min. The cells were washed for another three times to remove fluorescence probe and suspended in cell culture medium without phenolsulfonphthalein. Fluorescence intensity of HepG2 cells was determined by Gallios™ Flow Cytometer (Beckman Coulter, USA).

### 2.13. Detection of Mitochondrial Membrane Lipid Peroxidation

Mitochondrial lipid peroxidation was determined as previously described [14]. In brief, HepG2 cells were incubated with EC (60 mM) for 24 h in the presence or absence of MFE (0.5 mg/mL, 1 mg/mL, and 2 mg/mL). After incubation, the cells were washed and incubated for 30 min at 37°C with 10 *μ*M NAO. At last, the cells were washed thrice with PBS and immediately observed under fluorescence microscope. The results were expressed as mean NAO fluorescence intensity.

### 2.14. Statistical Analysis

All data are expressed as mean ± standard deviation (SD) from at least three independent experiments and analyzed by one-way ANOVA using SPSS (version 19.0). *p* < 0.05 was considered statistically significant.

## 3. Results and Discussion

### 3.1. Analysis of Phenolic Compounds in Mulberry Fruit Extract (MFE)

**Figure 1:**
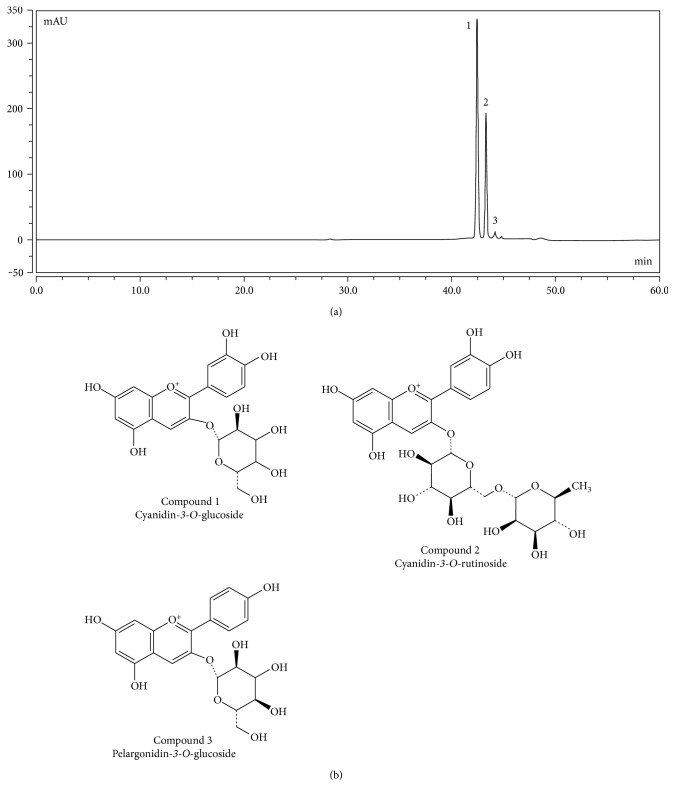
HPLC chromatograms of anthocyanins in MFE at 520 nm. (a) Chromatogram of anthocyanins at 520 nm. (b) Chemical structures of three anthocyanins.

Berry fruits are known to be rich in phenolic compounds such as phenolic acids, flavonoids, and anthocyanins, and various biological activities like antioxidant activity are associated with these phenolic compounds. Therefore, total phenolic content and total flavonoid content in mulberry fruit extract (MFE) were first determined. As shown in Table 1, total phenolic content was 502.43 ± 5.10 mg GAE/100 g FW and total flavonoid content was 219.12 ± 4.45 mg QE/100 g FW. Compared with some berry fruits like strawberry [21], blackberry [21], blueberry [22], and raspberry [23], the content of phenolic compounds in mulberry fruits was much higher. As a result, mulberry fruits can be considered as a good source for dietary supplement of phenolic compounds. In the present study, the moisture of raw material of mulberry fruit extract (MFE) was 86.22 ± 0.11%. Anthocyanin, as a type of phenolic compounds, possesses a potent antioxidant activity. In the present study, the category and content of anthocyanins in MFE were identified and measured by HPLC with the relevant standards. In Figure 1, three peaks were shown in the chromatography at 520 nm; they were identified as cyanidin*-3-O-*glucoside (peak 1), cyanidin*-3-O-*rutinoside (peak 2), and pelargonidin*-3-O-*glucoside (peak 3) based on the corresponding standards and published data [24]. Their contents were 81.36 ± 2.05 mg/100 g FW, 36.05 ± 1.14 mg/100 g FW, and 12.46 ± 0.65 mg/100 g FW in sequence. As we can see, cyanidin*-3-O-*glucoside and cyanidin*-3-O-*rutinoside occupied a large proportion of total anthocyanins in MFE. According to our results, it can be concluded that MFE was rich in phenolic compounds, which may endow it with a potent antioxidant activity.

### 3.2. Antioxidant Activity of MFE

Followed by determination of phenolic compounds of MFE, we next studied the antioxidant activity of MFE. Since MFE is rich in different kind of polyphenols, a single method for determining antioxidant activity cannot give a comprehensive prediction of antioxidant capacity. Therefore, three *in vitro* assays such as DPPH assay, ABTS assay, and FRAP assay were used to determine the antioxidant activity of MFE based on different mechanisms [25], and vitamin C was used as positive control. As we can see from Table 2, based on the capacity to reduce ferric ions to ferrous form (FRAP assay), MFE showed an appreciable ferric reducing capacity (2.54 ± 0.08 mg VCE/g FW), indicating that it has a potential antioxidant activity. Followed by FRAP assay, we further analysed the antioxidant activity of MFE using DPPH and ABTS assay. In line with ferric reducing capacity, MFE exhibited a similar antioxidant capacity (2.45 ± 0.15 mg VCE/g FW) to scavenge DPPH radical, while for ABTS assay, the antioxidant activity per gram of mulberry fruits is equal to 3.69 ± 0.05 mg VCE, demonstrating that ABTS radical was more sensitive than DPPH radical or ferric ions when exposed to MFE. Combining with the aforementioned results, it maybe concluded that the presence of abundant phenolic compounds in MFE contributed to their antioxidant potential. Indeed, phenolic compounds are composed of one or more aromatic rings possessing one or more hydroxyl groups and are therefore capable of quenching free radicals by forming resonance-stabilized phenoxyl radicals [26].

### 3.3. Protective Role of MFE on EC-Induced Cytotoxicity in HepG2 Cells

**Figure 2:**
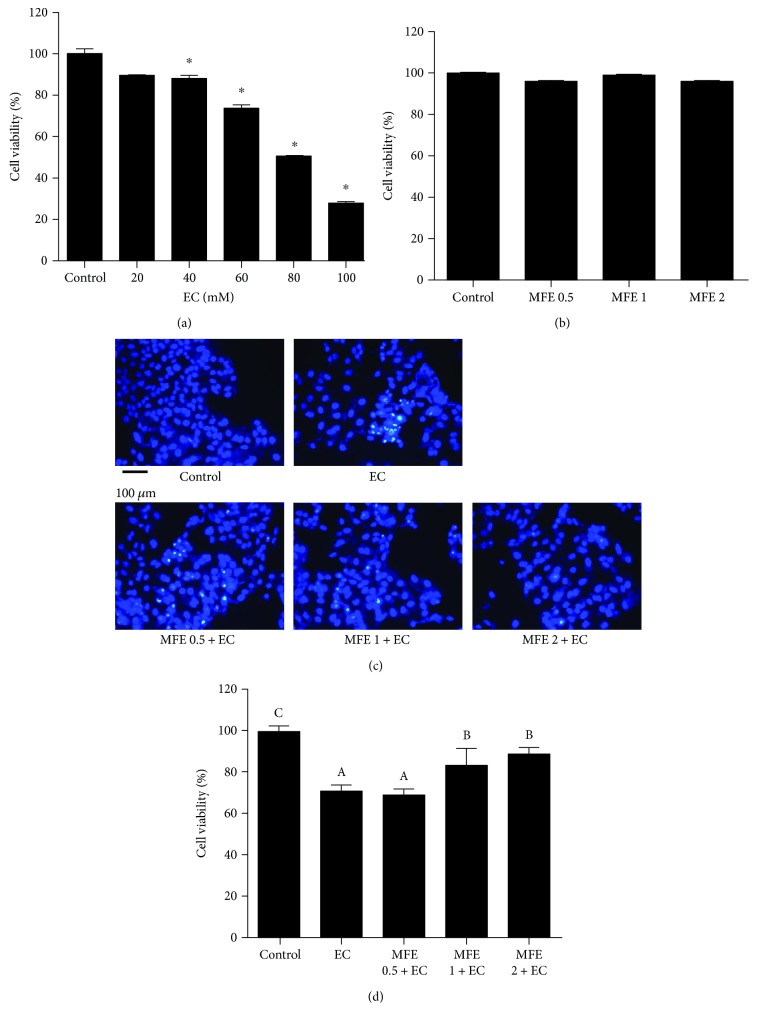
Effect of MFE on EC-induced toxicity in HepG2 cells. (a) The quantitative data of cell viability; HepG2 cells were treated with different concentrations of EC ranging from 0 to 100 mM for 24 h. (b) The quantitative data of cell viability; HepG2 cells were treated with different concentrations of MFE (0.5 mg/mL, 1 mg/mL, and 2 mg/mL) for 24 h. (c) Nuclear staining of HepG2 cells with Hoechst 33258. (d) The quantitative data of cell viability; HepG2 cells were incubated with 60 mM EC for 24 h in the presence or absence of MFE (0.5 mg/mL, 1 mg/mL, and 2 mg/mL). Results were expressed as mean value ± standard deviations. ^∗^*p* < 0.05 represents significant difference compared with EC group. EC, ethyl carbamate; MFE, mulberry fruit extract.

Human liver HepG2 cells were employed to investigate the protective effect of MFE against EC-induced cytotoxicity. As shown in Figures 2(a) and 2(b), HepG2 cells incubated with EC alone showed a decrease in cell viability in a concentration-dependent manner, whereas no decrease in cell viability was observed with the MFE treatment (0.5 mg/mL, 1 mg/mL, and 2 mg/mL) alone as compared with the control (cell viability defined as 100%). On the basis of MTT results, concentration of 60 mM of EC and three gradient concentrations (0.5 mg/mL, 1 mg/mL, and 2 mg/mL) of MFE were used in subsequent experiments. In order to evaluate the protective role of MFE, HepG2 cells were pretreated with MFE for 24 h and then incubated with 60 mM of EC for another 24 h, followed by MTT assay. As shown in Figure 2(c), compared with the cell viability of control group, cell viabilities of other groups were 71.18% ± 2.27% (EC-treated group), 83.61% ± 7.64% (1 mg/mL of MFE-treated group), and 89.18% ± 2.49% (2 mg/mL of MFE-treated group), indicating that MFE treatment (1 mg/mL and 2 mg/mL) afforded a protection against EC-induced cytotoxicity. In comparison with our previous study [5, 6], MFE is stronger than raspberry extract and blackberry extract in providing protection against EC-induced toxicity. Previous findings revealed that EC could be metabolised to its highly active epoxide form like vinyl carbamate epoxide, which could form DNA adducts eventually leading to DNA damage [27]. Therefore, we investigated whether MFE pretreatment could protect HepG2 cells from EC-induced genotoxicity. Hoechst 33258, a fluorescence probe which can sensitively bind to adenine-thymine sites of DNA, was used for evaluating EC-induced genotoxicity. As seen from Figure 2(d), EC-induced genotoxicity was observed based on the proportion of nuclear staining in cells treated with EC alone compared with control and MFE pretreated cells. Nucleus in more number of cells was visualised with bright blue dots in cells treated with EC alone indicating DNA damage where chromatin condensation or nuclear fragmentation were involved, whereas HepG2 cells pretreated with MFE showed a decrease in number of cells with bright blue dots demonstrating that MFE pretreatment afforded protection against EC-induced cytotoxicity in HepG2 cells.

### 3.4. MFE Afforded Protection against EC-Induced ROS Production

**Figure 3:**
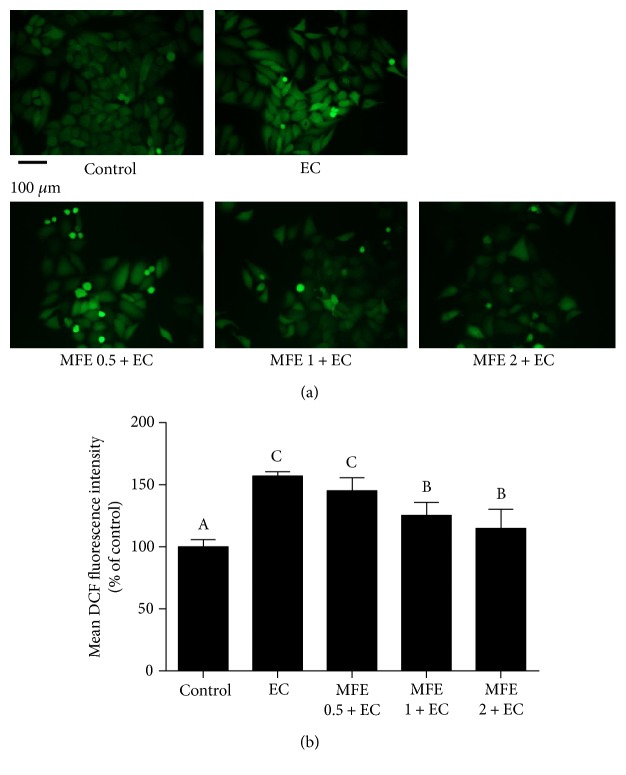
Effect of MFE on EC-induced ROS production in HepG2 cells. (a) Cells were pretreated with different concentrations of MFE (0.5 mg/mL, 1 mg/mL, and 2 mg/mL) for 24 h and then incubated with EC for another 24 h. After that, cells were collected and incubated with 10 *μ*M of DCFH-DA at 37°C for 30 min, then washed with PBS and evaluated by fluorescence microscope. (b) The quantitative data of panel (a) and results were expressed as mean DCF fluorescence intensity ± standard deviations. Different letters represent significant difference (*p* < 0.05). EC, ethyl carbamate; MFE, mulberry fruit extract; DCFH-DA, 2′,7′-dichlorofluorescin diacetate.

Increasing evidences depicted that cytotoxicity induced by exogenous substances was related to oxidative stress [28]. This further intrigued us whether EC-induced cytotoxicity was associated with overproduction and accumulation of ROS. Therefore, the level of cellular ROS in HepG2 cells followed by EC-incubation was determined by DCF fluorescence assay. As shown in Figures 3(a) and 3(b), the level of cellular ROS in HepG2 cells incubated with EC alone was significantly increased with the mean DCF fluorescence intensity reaching to 157.10% ± 2.35%, indicating that ROS was overproduced and accumulated after EC incubation. Interestingly, pretreatment with 1 mg/mL of MFE and 2 mg/mL of MFE make the mean DCF fluorescence intensity recover to 125.42% ± 9.53% and 114.83% ± 14.39%, respectively, which demonstrated that overproduced ROS was scavenged after MFE incubation. This finding suggested that EC-induced cytotoxicity was associated with overproduction of ROS and MFE afforded protection against EC-induced cytotoxicity via preventing ROS overproduction and accumulation in HepG2 cells.

### 3.5. MFE Inhibited Glutathione Depletion in HepG2 Cells

**Figure 4:**
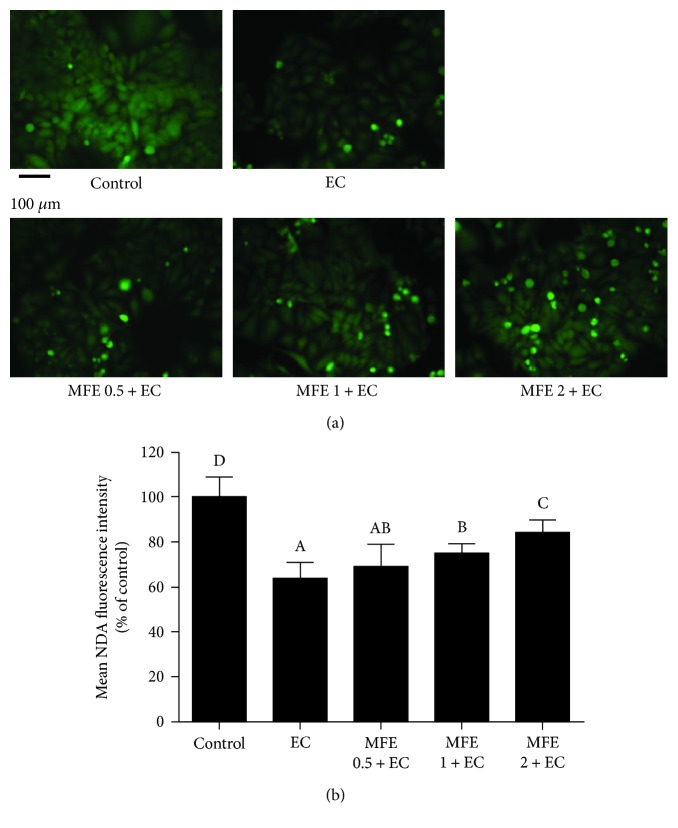
Effect of MFE on EC-induced GSH depletion in HepG2 cells. (a) Cells were pretreated with different concentrations of MFE (0.5 mg/mL, 1 mg/mL, and 2 mg/mL) for 24 h and then incubated with EC for another 24 h. After that, cells were collected and incubated with 50 *μ*M of NDA at 37°C for 30 min then washed with PBS and evaluated by fluorescence microscope. (b) The quantitative data of panel (a) and results were expressed as mean NDA fluorescence intensity ± standard deviations. Different letters represent significant difference (*p* < 0.05). EC, ethyl carbamate; MFE, mulberry fruit extract; GSH, glutathione; NDA, naphthalene-2.

Glutathione (GSH) as one of the most abundant cellular antioxidants plays an important role in maintaining cellular redox balance [29]. Taking this into consideration, we evaluated whether EC incubation could cause GSH depletion in HepG2 cells and investigated the protective effect of MFE on GSH depletion by NDA fluorescence probe. As shown in Figures 4(a) and 4(b), the NDA-stained cells were considerably reduced (mean NDA fluorescence intensity is 64.04% ± 6.04%) where cells were incubated with EC alone. However, the NDA staining was significantly increased both in 1 mg/mL of MFE-treated cells and 2 mg/mL of MFE-treated cells (mean NDA fluorescence intensity were 74.56% ± 4.74% and 84.18% ± 5.10%, resp.), suggesting that EC-induced cytotoxicity was associated with GSH depletion, and MFE could restore the intracellular GSH thereby providing strength to maintain cellular redox balance.

### 3.6. MFE Prevented EC-Induced Mitochondrial Membrane Damage

#### 3.6.1. MFE Abrogated EC-Induced Mitochondrial Membrane Potential Collapse

**Figure 5:**
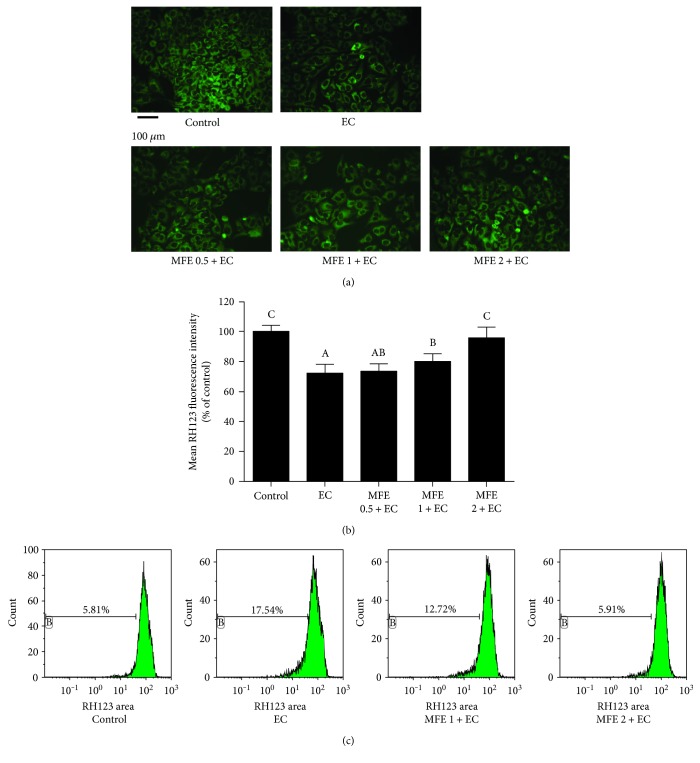
Effect of MFE on EC-induced mitochondrial membrane potential (MMP) decrease in HepG2 cells. (a) Cells were pretreated with different concentrations of MFE (0.5 mg/mL, 1 mg/mL, and 2 mg/mL) for 24 h and then incubated with EC for another 24 h. After that, cells were collected and incubated with 10 *μ*M of Rh123 at 37°C for 30 min and then washed with PBS and evaluated by fluorescence microscope. (b) The quantitative data of panel (a) and results were expressed as mean Rh123 fluorescence intensity ± standard deviations; different letters represent significant difference (*p* < 0.05). (c) Cells were pretreated with 1 mg/mL or 2 mg/mL of MFE and then incubated with EC for 24 h, followed by incubation with 10 *μ*M of Rh123, and after that, MMP was analysed by flow cytometry. Data represent similar results from three independent experiments. EC, ethyl carbamate; MFE, mulberry fruit extract; Rh123, rhodamine 123.

Mitochondria are the major site for ROS generation, and overproduction of ROS is related to MMP collapse [30]. The above findings make us explore whether the protective effect of MFE against EC-induced cytotoxicity was associated with MMP collapse. Rhodamine 123 (Rh123), a lipophilic cationic dye, can bind to the inner mitochondrial membrane followed by entering into the mitochondria, and further, it is retained within the mitochondria based on the negative MMP [20]. Therefore, we next determined MMP using Rh123. As shown in Figures 5(a) and 5(b), Rh123 fluorescence intensity (mean fluorescence intensity is 71.71% ± 5.83%) was markedly declined after EC treatment compared to that in the control group (fluorescent intensity of 100%), indicating that the decrease in MMP was followed by treatment with EC alone. However, MFE pretreatment (2 mg/mL) significantly prevented EC-induced decline in MMP where the mean fluorescence intensity reached to 95.60% ± 6.98%.

Followed by fluorescence microscopic detection of MMP collapse, flow cytometry combined with Rh123 staining was further used to confirm EC-induced MMP change. As expected, compared with control group, the percentage of cells with loss of MMP increased from 5.81% to 17.54% after EC treatment, which indicated that EC could cause a significant decrease of MMP. Similarly, when pretreated with 1 mg/mL and 2 mg/mL of MFE for 24 h, the percentage of cells with loss of MMP decreased to 12.72% and 5.91%, respectively (Figure 5(c)). These results jointly demonstrated that MFE could effectively ameliorate EC-induced MMP decrease.

#### 3.6.2. MFE Inhibited EC-Induced Mitochondrial Membrane Lipid Peroxidation

**Figure 6:**
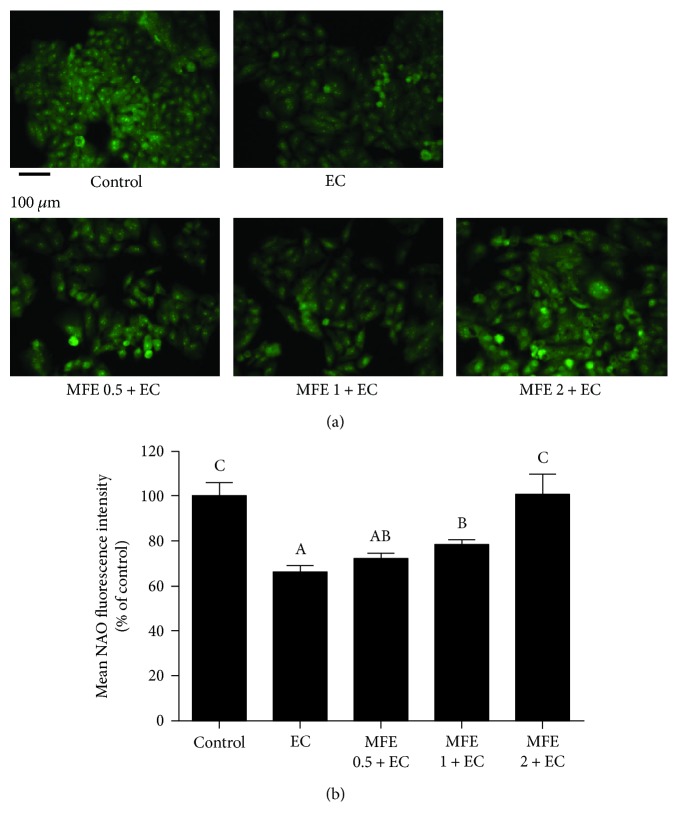
Effect of MFE on EC-induced mitochondrial membrane lipid peroxidation in HepG2 cells. (a) Cells were pretreated with different concentrations of MFE (0.5 mg/mL, 1 mg/mL, and 2 mg/mL) for 24 h and then incubated with EC for another 24 h. After that, cells were collected and incubated with 10 *μ*M of NAO at 37°C for 30 min and then washed with PBS and evaluated by fluorescence microscope. (b) The quantitative data of panel (a) and results were expressed as mean NAO fluorescence intensity ± standard deviations. Different letters represent significant difference (*p* < 0.05). EC, ethyl carbamate; MFE, mulberry fruit extract; GSH, glutathione; NAO, nonyl acridine orange.

Another disruptive effect of ROS in the mitochondria is membrane lipid peroxidation [31]. Oxidation of mitochondrial lipid membranes could lead to formation of reactive lipid electrophiles; therefore, lipid peroxidation induced by ROS is linked with altered redox status in hepatocellular carcinoma [32]. Since EC treatment declined the MMP followed by overproduction of ROS as observed in aforementioned results, we therefore examined whether EC is able to induce lipid peroxidation in mitochondria of HepG2 cells. NAO fluorescence probe was used to detect cardiolipin, a major mitochondrial membrane lipid component which undergoes oxidative degradation in the presence of ROS [20]. HepG2 cells treated with EC alone showed a remarkable decrease in mean fluorescence intensity (65.83% ± 3.03%) compared with those in the control group (100%). In contrast, the cells pretreated with 1 mg/mL or 2 mg/mL of MFE showed a significant increase in mean NAO fluorescence intensity. The protective effect of 2 mg/mL of MFE on inhibiting EC-induced mitochondrial membrane lipid peroxidation was equal to that of the control group (Figures 6(a) and 6(b)). Our results suggested that MFE exhibited a good performance in inhibiting EC-induced oxidative damage to mitochondria.

## 4. Conclusion

In summary, the present study unveiled that polyphenolic-rich MFE was able to exert potential protective effect against ethyl carbamate-induced cytotoxicity. Our results showed that MFE was rich in polyphenols with total phenolic content and total flavonoid content reaching to 502.43 ± 5.10 and 219.12 ± 4.45 mg QE/100 g FW, respectively. Further HPLC analysis indicated that cyanidin*-3-O-*glucoside and cyanidin*-3-O-*rutinoside were the major anthocyanins. *In vitro* studies related to antioxidant activity (DPPH, ABTS, and FRAP assays) indicated that MFE is a source of efficacious antioxidants. In addition, MFE was found to possess a potent ability to protect human liver HepG2 cells from EC-induced cytotoxicity via scavenging overproduced cellular ROS. More importantly, our results proved that the protective effect of MFE against EC-induced cytotoxicity was through inhibiting GSH depletion, MMP collapse, and mitochondrial membrane lipid peroxidation. In conclusion, our results suggested that mulberry fruit extract is able to afford protection against EC-induced cytotoxicity and oxidative stress via its antioxidant nature.

## Figures and Tables

**Table 1:** Composition and content of phenolic compounds in the mulberry fruit extract (MFE).

	Total phenolics (mg GAE/100 g FW)	Total flavonoids (mg QE/100 g FW)	Anthocyanins (mg/100 g FW)		
			C3G	C3R	P3G
Mulberry	502.43 ± 5.10	219.12 ± 4.45	81.36 ± 2.05	36.05 ± 1.14	12.46 ± 0.65

The values are expressed as mean ± SD (*n* = 3). C3G: cyanidin*-3-O-*glucoside; C3R: cyanidin*-3-O-*rutinoside; P3G: pelargonidin*-3-O-*glucoside; FW: fresh weight; GAE: gallic acid equivalents; QE: quercetin*-3-O-*rutinoside equivalents.

**Table 2:** Antioxidant activity of MFE.

	ABTS (mg VCE/g FW)	DPPH (mg VCE/g FW)	FRAP (mg VCE/g FW)
Mulberry	3.69 ± 0.05	2.45 ± 0.15	2.54 ± 0.08

The values are expressed as mean ± SD (*n* = 3); VCE: vitamin C equivalents; FW: fresh weight.
